# Not going with the flow: Locomotor activity does not constrain immunity in a wild fish

**DOI:** 10.1002/ece3.5658

**Published:** 2019-10-02

**Authors:** Numair Masud, Rebecca Synnott, Pascal I. Hablützel, Ida M. Friberg, Joanne Cable, Joseph A. Jackson

**Affiliations:** ^1^ School of Biosciences Cardiff University Cardiff UK; ^2^ School of Environment and Life Sciences University of Salford Salford UK; ^3^ IBERS Aberystwyth University Aberystwyth UK; ^4^ Flanders Marine Institute Oostende Belgium; ^5^ Laboratory of Biodiversity and Evolutionary Genomics Biology Department University of Leuven Leuven Belgium

**Keywords:** gene expression, *Gyrodactylus*, immunity, physical activity, rheotaxis, stickleback

## Abstract

Immunity is a central component of fitness in wild animals, but its determinants are poorly understood. In particular, the importance of locomotory activity as a constraint on immunity is unresolved. Using a piscine model (*Gasterosteus aculeatus*), we combined a 25‐month observational time series for a wild lotic habitat with an open flume experiment to determine the influence of locomotor activity (countercurrent swimming) on natural variation in immune function. To maximize the detectability of effects in our flume experiment, we set flow velocity and duration (10 cm/s for 48 hr) just below the point at which exhaustion would ensue. Following this treatment, we measured expression in a set of immune‐associated genes and infectious disease resistance through a standard challenge with an ecologically relevant monogenean infection (*Gyrodactylus gasterostei*). In the wild, there was a strong association of water flow with the expression of immune‐associated genes, but this association became modest and more complex when adjusted for thermal effects. Our flume experiment, although statistically well‐powered and based on a scenario near the limits of swimming performance in stickleback, detected no countercurrent swimming effect on immune‐associated gene expression or infection resistance. The field association between flow rate and immune expression could thus be due to an indirect effect, and we tentatively advance hypotheses to explain this. This study clarifies the drivers of immune investment in wild vertebrates; although locomotor activity, within the normal natural range, may not directly influence immunocompetence, it may still correlate with other variables that do.

## Graphical abstract

Immunity is a central component of fitness in wild animals, but its determinants are poorly understood. Using a piscine model (*Gasterosteus aculeatus*), we combined a 25‐month observational time series for a wild lotic habitat with an open flume experiment to determine the influence of locomotor activity (countercurrent swimming) on natural variation in immune function. This study clarifies the drivers of immune investment in wild vertebrates; although locomotor activity, within the normal natural range, may not directly influence immunocompetence, it may still correlate with other variables that do.

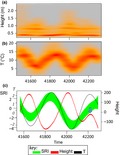

## 1 INTRODUCTION

In this study, we examine the consequences of locomotor activity for immunity in a model wild vertebrate, the three‐spined stickleback, *Gasterosteus aculeatus*. Like most animals, sticklebacks need to undertake locomotor activity to survive. In particular, individuals living in flowing water must maintain station within suitable habitat through countercurrent swimming (rheotaxis) (Arnold, 1974). This expends energy and may functionally interfere with other physiological processes (Kieffer, 2000), perhaps altering immune allocation and function (van Dijk & Matson, 2016). Alteration in immunity, in turn, is likely to affect health and fitness at the individual and population level—influencing the development of disease within individuals (Parkin & Cohen, 2001) and constraining the transmission of infectious disease between individuals (Hellriegel, 2001). Thus, being able to understand and predict sources of variation in immune function will often be necessary to understand the dynamics of disease. Moreover, despite increasing recognition that immune variation is generated largely by environmental effects, perhaps including locomotory responses to the environment, the sources of this variation are poorly understood, even in humans and laboratory mice (Beura et al., 2016; Brodin et al., 2015).

Physical activity is widely thought to influence the immune system (Pedersen & Hoffman‐Goetz, 2000; Walsh et al., 2011), and, furthermore, has often been considered to exert suppressive, generally transient, effects that increase disproportionately at more extreme levels of activity (van Dijk & Matson, 2016; Nieman, 2000). Nonetheless, the latter paradigm has also been challenged and immunological changes following intense exercise interpreted differently—as a beneficial heightening of immune surveillance and regulation (Campbell & Turner, 2018). The evidence for these contrasting paradigms in naturally occurring vertebrates is even less clear‐cut and largely derived from a limited number of studies in birds, with fewer studies in other vertebrate classes (Brown & Shine, 2014; Husak, Ferguson, & Lovern, 2016). In birds, flight experiments (Matson, Horrocks, Tieleman, & Haase, 2012; Nebel et al., 2012; Nebel, Buehler, MacMillan, & Guglielmo, 2013) mostly suggest immunosuppressive effects of sustained flight, but contrary observations of no effect (Hasselquist, Lindstrom, Jenni‐Eiermann, Koolhaas, & Piersma, 2007) have also been reported.

Through integrating field observation with matched experimental manipulation of sticklebacks, the present study aims to place the effects of water flow, and associated countercurrent swimming, within the context of overall environmental effects on immunity in the wild. To achieve this, we consider both field records of water flow and immune gene expression in a lotic habitat and effects estimated in an experiment in which acclimated fish were made to swim in an open flume under controlled conditions. Our design is intended, as far as possible, to avoid the artificiality of laboratory models and the very weak inference typically possible in purely observational studies (due to collinearity and confounding of variables).

For the field component of our study, we analyzed a 25‐month time series, containing monthly expression data for immune‐associated genes and fine‐scale thermal and flow data (all of which showed strong sinusoid‐like circannual oscillation). For our flume experiment, we used effectively wild fish (from a naturalistic, parasite‐exposed habitat) that had first been treated to remove parasites and acclimatized. As immune system expression is likely to depend on past individual experience of the environment, this ensured that our experimental subjects had a history of natural environmental exposures and a relatively natural immunophenotype. (In contrast, laboratory‐bred subjects would have had past environmental exposures very different to those in the wild, and very different immunophenotypes, unrepresentative of those in nature).

In both the field and experiment, we used a standard set of gene expression measurements that we have previously demonstrated to precisely report a dominant genome‐wide seasonal oscillation in immune‐associated gene expression in wild sticklebacks (Brown et al., 2016; Stewart, Hablutzel, et al., 2018). This oscillation corresponds to experimentally determined infection resistance (Stewart, Hablützel, et al., 2018; Stewart, Hablutzel, et al., 2018) and is partly driven directly by environmental temperature and partly by other, as yet unidentified, seasonal environmental variation that might include variation in flow effects (Stewart, Hablützel, et al., 2018; Stewart, Hablutzel, et al., 2018). Seasonal progression explains more genome‐wide variation in immune‐associated gene expression than other relevant factors (including geographic site, sex, and ontogeny) and is characterized by outlying expression values in the late winter and late summer (Brown et al., 2016). For simplicity, as previously described (Stewart, Hablutzel, et al., 2018), we are able to combine our set of gene expression measurements into a single representative index (seasonal reporter index, SRI). Moreover, to cross‐reference gene expression variation and our experimental treatments to a functional phenotype, we also directly measured infection resistance in our flow experiment. This was based on challenge infections with the ecologically relevant (pathogenic and naturally occurring) monogenean ectoparasite, *Gyrodactylus gasterostei* (Stewart et al., 2017), a directly transmitted viviparous species that proliferates in situ on the host skin.

Our study was thus designed to allow us to partition the effects of locomotory activity from other sources of variation in immune allocation in nature and to quantify them. In the event, we found no effect of sustained and intense countercurrent swimming, suggesting that variation in locomotory activity has a negligible direct influence on immune allocation and function in wild fish. Nonetheless, we did find that seasonal flow rates were correlated with immune allocation in the wild and so we further consider below possible indirect effects of water flow on seasonal immune variation.

## 2 MATERIALS AND METHODS

### 2.1 Field site and field observations

The study site (RHD, 52.4052, −4.0372) was a small area in an oligotrophic, fully freshwater, side‐channel of the River Rheidol. As the Rheidol traverses an unusually steep gradient and is subject to anthropogenic water releases from the Cwm Rheidol dam (upstream of the study site), it experiences a very variable flow regimen, with maximum flows above 30 m/s (Whoriskey & Wootton, 1987) possible, even in side channels. The site held a large population of sticklebacks with an approximately annual life history (young of the year largely replacing the previous year cohort by early autumn) (Wootton, 2007). A calibrated TinyTag Aquatic 2 data logger was placed at a representative depth within the habitat, recording temperature readings every 5 min throughout the study. To provide information on water flow in the river, water level readings, taken every 15 min, were obtained from a gauge at Cwm Rheidol (data kindly supplied by National Resources Wales). Ten fish were sampled per month from RHD for 25 months between October 2013 and October 2015. Monthly data were missing for October and December 2014 due to inclement weather. Gene expression data for the RHD fish have previously been reported (Stewart, Hablutzel, et al., 2018). As previously described, fish were individually captured with dipnets and immediately killed by concussion and decerebration, then conserved in RNA stabilization solution and transferred to −80°C for long‐term storage (Brown et al., 2016). Standard length (tip of snout to tail fork, mm), weight (mg), and sex were recorded for all fish.

### 2.2 Open flume experiment

Sticklebacks, from a self‐propagating population in seminatural (lentic) outdoor fish ponds (Surrey, UK), were obtained in January 2017 and transported to Cardiff University. As the source population was exposed to a natural community of parasites, the fish were subject to antiparasitic treatment. Fish were first submerged in 0.004% formaldehyde solution for 1 hr, with a half‐hour rest period in between, and then maintained in 1% aquarium salt and 0.002 g/L methylene blue for 48 hr to prevent secondary infections. Subsequently, each individual fish was visually screened for ectoparasites three times by anesthetizing them in 0.02% MS‐222 and observing them using a dissecting microscope with fiber optic illumination. This screen involved removing any remaining ectoparasites with a watchmaker's forceps following the methods of (Schelkle, Shinn, Peeler, & Cable, 2009). After treatment, sticklebacks were maintained in 70 L tanks with dechlorinated water (temperature: 14 ± 1°C; photoperiod: 12 hr light/12 hr dark; approximating late summer conditions). Fish were fed daily, to satiation, on a diet of frozen bloodworms and acclimatized to laboratory conditions over a period of 8 weeks. The above temperature, lighting, and diet conditions were maintained throughout the experimental trials.

In employing fish from a lentic habitat for our experiment, we expected that these might be less preconditioned to sustained swimming and thus allow more sensitive detection of any locomotion effect on immune gene expression. We also note that natural variation in the gene expression readouts that we employ below is largely due to consistent environmental responses, with fish of different genetic identity in different habitats responding similarly to seasonal change (Brown et al., 2016; Stewart, Hablutzel, et al., 2018). Thus, differences in the genetic background of the study fish are expected to be unimportant.

The flow experiment was conducted in a perspex open channel recirculating flume (channel length: 150 cm, depth: 16 cm, and height: 20 cm), filled to 15 cm depth, to expose fish to water flow. To create flow, an impeller with a diameter of 10 cm was attached to a 1 horse power three phase 4‐pole motor with a maximum shaft speed of 1,500 rpm (Machine Mart) and wired to a 1.1 kW inverter (RS Components) which controlled motor speed. Aluminium honeycomb flow straighteners (width: 20 mm, cell diameter: 6.4 mm) were inserted at both ends of the flume to provide laminar flow, restricting the fish to a 100‐cm length section. Based on preliminary trials, a flow speed of 10 cm/s (25 Hz) was chosen for this experiment as it evoked countercurrent swimming (rheotaxis) without leading to exhaustion, defined as an inability to maintain station (Whoriskey & Wootton, 1987). An identical flume with static conditions was used as a control. Flow speed was measured by recording the time taken for a neutrally buoyant ball to flow 1 m downstream, averaged over 10 replicates. Fish were placed in the flume at a stocking density of five fish per flow trial, and the flume was run for 48 hr (equivalent to traveling 17.3 km).

**Figure 1:**
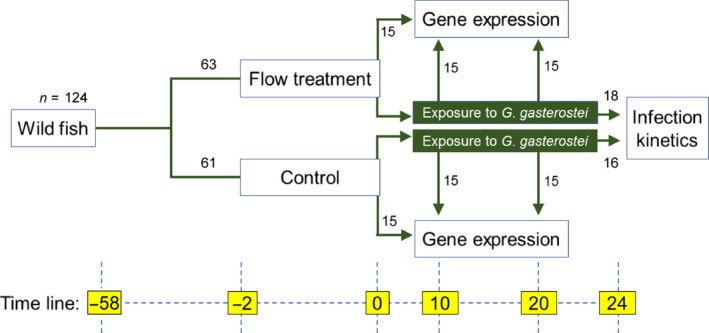
Schematic summarizing experimental design. Time (lower boxes) is expressed in days relative to the time of exposure with *Gyrodactylus gasterostei* (i.e., negative before parasite exposure)

To determine immune expression at the end of the 48 hr flow treatments (see Figure 1 for a summary of the experimental design), fish exposed to flow (*n* = 15) and control fish in static conditions (*n* = 15) were removed from the flume and immediately killed by an overdose of anesthetic (MS222) followed by decerebration (“baseline” fish). Further fish were removed from the flume and experimentally infected with two *Gyrodactylus gasterostei* worms as previously described (King & Cable, 2007). Briefly, a heavily infected donor stickleback was placed near a recipient fish and monitored under a dissecting microscope with fiber optic illumination until two worms were observed transferring to the caudal fins of the recipient (procedure lasting <5 min). Control fish, experiencing no‐flow conditions, were infected at the same time as flow‐treated fish. Infected fish were then maintained in 1 L containers, where a subset (*n* = 18 flow treatment; *n* = 16 control) were screened for parasites (full count of individuals) every 4 days (King & Cable, 2007) over a 24‐day infection trajectory (Figure 1). The remaining infected fish were sampled to determine immune expression (as above) at days 10 or 20 of the infection trajectory (*n* = 15 fish per treatment × time combination) (Figure 1). The viviparous in situ infrapopulation growth of *G. gasterostei*, on isolated fish at the temperatures used here, typically features an initial postinfection increase (up‐phase), followed by a peak and decrease (down‐phase) to zero or very low numbers (Harris, 1983). The 10‐ and 20‐day time points used above, respectively, correspond to the “up‐phase” and “down‐phase” of population growth in the present set of infections.

All fish killed at sampling points were immediately conserved in RNA stabilization solution, as previously described (Hablützel, Brown, Friberg, & Jackson, 2016) and transferred to −80°C for long‐term storage. Standard length, weight, and sex were recorded for all fish processed for gene expression and standard length for challenge infection fish. The range of standard length in experiment fish sampled for gene expression was 45–60 mm (compared with 15–61 mm in wild fish from RHD) (see Figure S1). The experiment fish were, on average, in poorer body condition than wild fish at RHD (Figure S1) (and there was no signification effect of flow treatment on condition measures, *P* > 0.05), suggesting that any relevant energy or nutrition‐mediated effect of locomotory activity on immune activity would not have been masked by an excessively high level of nutrition in the feeding regimen we employed.

### 2.3 Gene expression measurements

We quantified gene expression in RNA extracted from homogenized whole fish by reverse transcription quantitative real‐time PCR (QPCR) following previously described methods (Brown et al., 2016; Hablützel et al., 2016; Stewart, Hablutzel, et al., 2018). The advantages of using whole fish samples are considered in (Hablützel et al., 2016). We measured a set of five genes (seasonal reporter, SR, genes) whose expression we have shown to reflect a major immune‐system‐wide seasonal expression signature (Brown et al., 2016; Stewart, Hablutzel, et al., 2018). These include three genes expressed highly in summer: *cd8a* (Ensembl gene identifier: ENSGACG00000008945), *foxp3b* (ENSGACG00000012777), and *ighm* (ENSGACG00000012799); two genes expressed highly in winter: *orai1* (ENSGACG00000011865) and *tbk1* (ENSGACG00000000607). As noted above, gene expression data for the field study at RHD have previously been considered, and detailed methods reported, in (Stewart, Hablutzel, et al., 2018). Equivalent methods were used to generate the gene expression dataset for the present flume experiment. Briefly, RNA was extracted from whole fish samples preserved in RNA stabilization solution using the Isolate II RNA mini kit (Bioline). Whole individual fishes were homogenized in kit lysis buffer using a 5‐mm stainless steel bead (Qiagen, 69,989) in a Qiagen TissueLyser LT system and a standard aliquot of the homogenate passed through the manufacturer‐recommended protocol. RNA extracts were DNAse treated and converted to cDNA using the High‐Capacity RNA‐to‐cDNA™ Kit (ThermoFisher), according to manufacturer's instructions, including reverse transcription negative (RT‐) controls for a subsample. Assays were pipetted onto 384‐well plates by a robot (Pipetmax, Gilson) using a custom programand run on a QuantStudio 6‐flex Real‐Time PCR System (ThermoFisher) at the machine manufacturers default real‐time PCR cycling conditions. Reaction size was 10 µl, incorporating 1 µl of template and Applied Biosystems™ Fast SYBR™ Green Master Mix (ThermoFisher) and primers at the machine manufacturer's recommended concentrations. Samples from different experimental treatment groups were dispersed across three plates. Each plate contained all target gene expression assays and two endogenous control gene assays, for samples (in duplicate) and a calibrator sample (in triplicate). Endogenous control genes (*yipf4*, *acvlr1*) were previously validated (Brown et al., 2016), as a pairing, for stability under seasonal variation. Primers used were reported in Brown et al. (2016) and Hablützel et al. (2016). In addition, no template controls for each gene were included on each plate. Template cDNA (see above) was diluted 1/20 prior to assay. The calibrator sample (identical on each plate) was created by pooling cDNA derived from whole fish RNA extracts from wild sticklebacks captured in summer. Relative gene expression values used in analyses are RQ values calculated by the QuantStudio 6‐flex machine software according to the ∆∆Ct method, indexed to the calibrator sample. Melting curves and amplification plots were individually inspected for each well replicate to confirm specific amplification.

### 2.4 Parasite material

Parasites originated from a laboratory culture of *Gyrodactylus gasterostei* recently derived from wild sticklebacks at Roath Park, Cardiff (51.506, −3.175) and passaged on uninfected sticklebacks.

### 2.5 Ethics statement

All experimental work conducted at the Cardiff University aquatic laboratory was approved by the Cardiff University animal ethics committee and conducted under UK Home Office license PPL 303424. Field sampling was approved by the animal welfare committee of the Institute of Biological, Environmental and Rural Sciences (IBERS), Aberystwyth University.

### 2.6 Data analysis

The field and experiment gene expression datasets, respectively, contained 219 and 54 individuals with no missing values. In the experiment, only a random subsample of the fish sampled at day 10 and day 20 (6 per treatment group) were processed for gene expression (see Figure 1). The *G. gasterostei* challenge infection dataset from the experiment contained 34 individuals, 3 of which had missing standard lengths but no other missing values.

All analyses were carried out using *R* version 3.4.4 (R Core Team, 2018). For the field dataset, we combined our gene expression measurements into an additive index (seasonal reporter index, SRI) that we have previously shown to reflect a major pattern of seasonality in the expression of stickleback immune‐associated genes. For this, each raw relative gene expression variable was first log_10_ transformed and standardized. The values for each gene variable were then summed, assigning negative or positive values to genes according to whether they were most expressed in winter (negative) or in summer (positive) in the transcriptomic study of Brown et al. (2016) (i.e., *cd8a* + *foxp3b* + *ighm* − *tbk1* − *orai1*). High values of the SRI reflect high expression of genes linked to the adaptive arm of the immune system. For the field dataset, we do not present the variation in individual gene expression variables contributing to SRI here as we have previously considered this in detail elsewhere (Stewart, Hablutzel, et al., 2018) and the individual variables all correlate very strongly with SRI.

For the field dataset, we initially separately analyzed variation in flow and temperature in generalized additive models (GAMs) and variation in SRI in generalized additive mixed models (GAMMs) (Wood, 2006). The nonparametric smoother term in these models was used to flexibly represent temporal trends, without presupposing a particular relationship. The GAMs for flow and temperature contained a thin plate spline smoother for time as an explanatory term. The GAMMs for gene expression variables additionally contained fixed effects for length and sex (male/female), and random intercepts for assay plate. GAMs and GAMMs (with normal errors) were implemented using the *gam* command in the *mgcv* package, with the random component in the GAMMs represented as penalized regression terms. To test the association of flow with SRI, while accounting for the effects of temperature variation (of known causal importance (Stewart, Hablutzel, et al., 2018)), we constructed a further GAMM with SRI as the response. Initially, these were of the same form as the SRI model above, except that the nonparametric smoother for time was replaced by a linear term for temperature. An additional term (either linear or a smoother) for flow was then added to the model.

To obtain an overall test of the effects of flow treatment on gene expression in our experimental (flume) dataset, we initially applied a permutational multivariate analysis of variance based on a distance matrix (PERMANOVA‐DM) to our 5 (untransformed) gene expression variables (*adonis* command; package *vegan*). The full model included main effect factor terms for flow treatment (flow or no flow), *Gyrodactylus* infection stage (baseline, up‐phase and down‐phase), sex and assay plate. It also included main effect continuous terms for standard length (mm) and body condition (residual from a quadratic regression of weight on length) and an interaction term for flow treatment and *Gyrodactylus* infection stage. Terms were assessed for significance through a backward selection procedure whereby the *p* value for individual terms was determined by addition (last) to the full model. The least significant term was omitted, and then, the process repeated to obtain a minimal model with only significant terms. Results reported below are based on addition to a minimal model containing only significant terms. Secondarily, we also analyzed variation in SRI and each of the individual gene expression variables separately, employing linear mixed models (LMMs) (*lmer* command; package *lme4*). For these analyses, the individual gene expression variables were first power transformed (determined through a Box‐Cox procedure) and then standardized (zero mean, unit standard deviation). We constructed a separate LMM for each variable containing the same fixed terms as in the PERMANOVA‐DM (above). Additionally, each LMM contained random intercepts for flow trial (experiment batch) and RNA extraction batch. Fixed effects were tested via a backward selection procedure, and results below for flow treatment and experimental stage are presented when these terms were, respectively, added to a base model (containing all other significant fixed terms and terms for plate, flow trial and RNA extraction batch, regardless of significance). Other model selection strategies gave identical conclusions with regard to the effect of flow and infection stage.

To quantify the individual 25‐day *Gyrodactylus* infection trajectories (see above), we utilized area under the curve for worm counts (AUC^worm count^), peak worm count and the maximum intrinsic rate of increase (Birch, 1948), *r*, observed over any of the 4‐day observation periods. *r* was calculated from the relationship:

$$N_{t\;=}N_{0}e^{\mathrm{rt}}$$

where *N* is the worm count, *t* is time in days, and *e* is the base of the natural logarithm). These were analyzed as the response in general linear models (LMs; *lm* command) with flow treatment (flow or no flow) as a factor and fish standard length as a continuous explanatory term. Prior to analysis, AUC^worm count^ and peak worm count were optimally transformed by a Box‐Cox procedure.

Standard diagnostic plots were inspected for all additive (*gam.check*) and linear (*plot.lm*, *plot.merMod*) models to check their suitability.

## 3 RESULTS

### 3.1 Flow rate and immune allocation were seasonal and correlated in a wild lotic habitat

**Figure 2:**
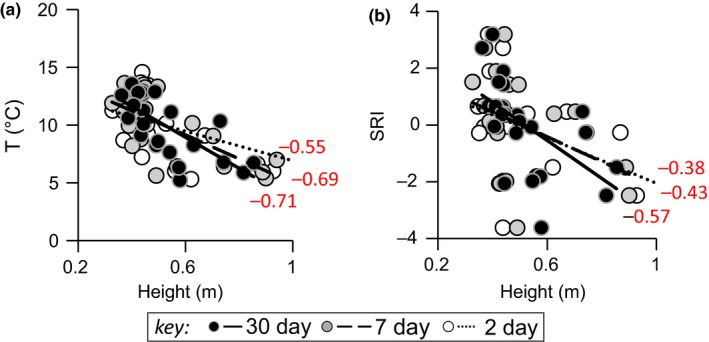
Crude correlation between water flow (Height, m), water temperature (*T*, °C) and immune‐associated gene expression (SRI) at the field site (RHD). Plots show mean water temperature (a) and monthly mean SRI (b) against mean water flow. The plotted data for temperature and flow are means for 2, 7, and 30 days prior to the monthly sampling point for SRI (see key). Figures on the plots are Pearson correlation coefficients. SRI (seasonal reporter index, SRI) is an additive index based on the expression of five separate genes (seasonal reporter genes, SR) known to reflect a consistent major seasonal oscillation in immune‐associated gene expression in wild stickleback (Brown et al., 2016; Stewart, Hablutzel, et al., 2018)

**Figure 3:**
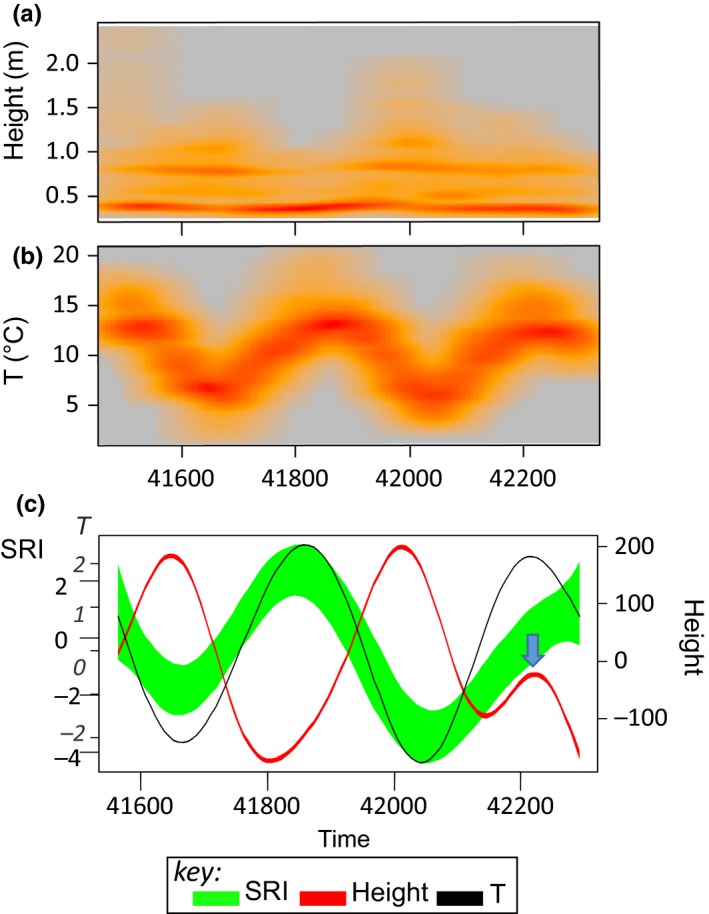
Temporal variation in water flow (Height, m), temperature (*T*, °C), and immune‐associated gene expression (SRI) at the field site (RHD). (a) Water height at a gauge upstream of the study locality plotted against time and shown as a smoothed color density representation obtained through a (2D) kernel density estimate; based on recordings taken every 15 min. (b) Water temperature (*T*) plotted against time and shown as a color density representation (see above), based on recordings taken every 5 min. (a–b) Increasing density of observed points is indicated by yellow to red colors. (c) 95% confidence intervals for (centered) nonparametric smoothers against time for water flow (red), water temperature (black) and immune‐associated gene expression, SRI (green). SRI (seasonal reporter index) is an additive gene expression index based on five genes (seasonal reporter genes, SR) known to report a dominant immunome‐wide seasonal oscillation; high values reflect high expression of genes involved in adaptive immunity (Brown et al., 2016; Stewart, Hablutzel, et al., 2018). On all plots, time is shown as days since 1 January 1900 (a standard format used by many computer programs); 41,600 = 22 November 2013; 42,200 = 15 July 2015. Note: later in the time series smoothed flow, unlike smoothed temperature, does not fully conform to a simple circannual sinusoid (with a late additional peak in the second year; arrow (c)) and throughout flow is more intermittent in character than temperature (a, b), meaning these environmental variables sometimes vary orthogonally

**Figure 4:**
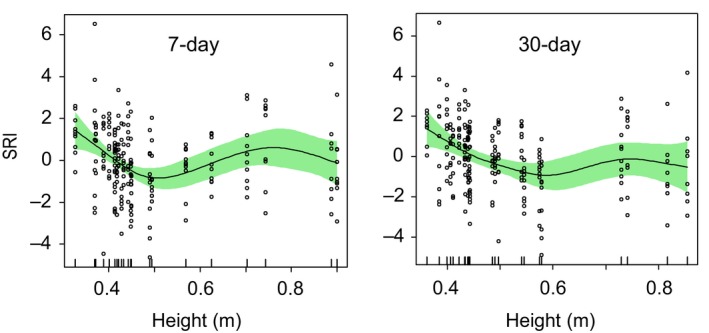
Confounder‐adjusted association of immune‐associated gene expression (SRI) with water flow (Height, m). Plotted lines are nonparametric smoothers (centered) from generalized additive mixed models (GAMMs) on the scale of the model linear predictor, with 95% confidence intervals shaded; scatter of points shows partial residuals. Analyses based on mean flow in the 7 and 30 days prior to the monthly sampling points for SRI. SRI (seasonal reporter index, SRI) is an additive index based on the expression of five separate genes (seasonal reporter genes, SR) known to reflect a consistent major seasonal oscillation in immune‐associated gene expression in wild stickleback (Brown et al., 2016; Stewart, Hablutzel, et al., 2018)

In our 25‐month time series from a lotic habitat (RHD), there was a strong crude correlation between water flow, water temperature, and SRI (which reflects high adaptive immune activity at high values) (Figure 2). All three of these variables showed sinusoid‐like circannual variation: water flow (winter peak) tending to vary in antiphase to SRI and temperature (summer peaks) (Figure 3). We have previously demonstrated experimentally that temperature drives SRI variation, with an effect size sufficient to account for a substantial part of the circannual oscillation in SRI at RHD (Stewart, Hablutzel, et al., 2018). We thus first asked whether flow could explain any SRI variation over and above that explained by temperature in a conventional linear statistical model. While bearing in mind that collinearity would likely wholly or partly mask any separate effects of temperature and flow, we also noted that flow was often irregular (due to irregular rainfall and anthropogenic dam release; see Figure 3) and that this might generate sufficient orthogonality with temperature variation to establish an independent association of flow and SRI (if not an interpretable effect size). We therefore constructed a GAMM explaining SRI in terms of confounder variables (sex and length) and temperature, and we then added flow as a further explanatory term to this base model. Flow was nonsignificant as a linear term, but significant when added to the model as a nonlinear nonparametric smoother, explaining a modest additional amount (up to 3.7 percentage points) of model deviance (Table 1, Figure 4).

**Table 1:** Results of a generalized additive mixed model (GAMM) explaining variation in immune‐associated gene expression in a 25‐month time series from a wild lotic habitat (RHD)

Base model	% Dev	Flow term	*P*	% Dev
Sex + *L* + temperature + *assay plate*	53.6	2 day (linear)	ns	53.6
		7 day (linear)	ns	53.5
		30 day (linear)	ns	53.4
		2 day (smoother)	ns	55.2
		7 day (smoother)	7.9 × 10^–4^	57.5
		30 day (smoother)	0.001	57.3

Note

The gene expression response variable is an additive index (seasonal reporter index, SRI) based on the expression of five separate genes (seasonal reporter genes, SR) known to reflect a consistent major seasonal oscillation in immune‐associated gene expression in wild stickleback (Brown et al., 2016; Stewart, Hablutzel, et al., 2018). Different terms representing flow (height, m) were added separately to a base model for SRI already containing fixed terms for sex, standard length (*L*, mm), and temperature (°C) and random intercepts for assay plate. Flow data were based on averages over 2, 7, and 30 days prior to the gene expression sample point and terms for these added to models were either linear or nonparametric smoothers. The percentage of total deviance explained (%Dev) is shown for the different models, with the best smoother model explaining only an additional 3.7 percentage points of the total deviance above the 53.6% explained by the base model.

### 3.2 Sustained intense swimming in an open flume had no effect on immune allocation or infection susceptibility

**Figure 5:**
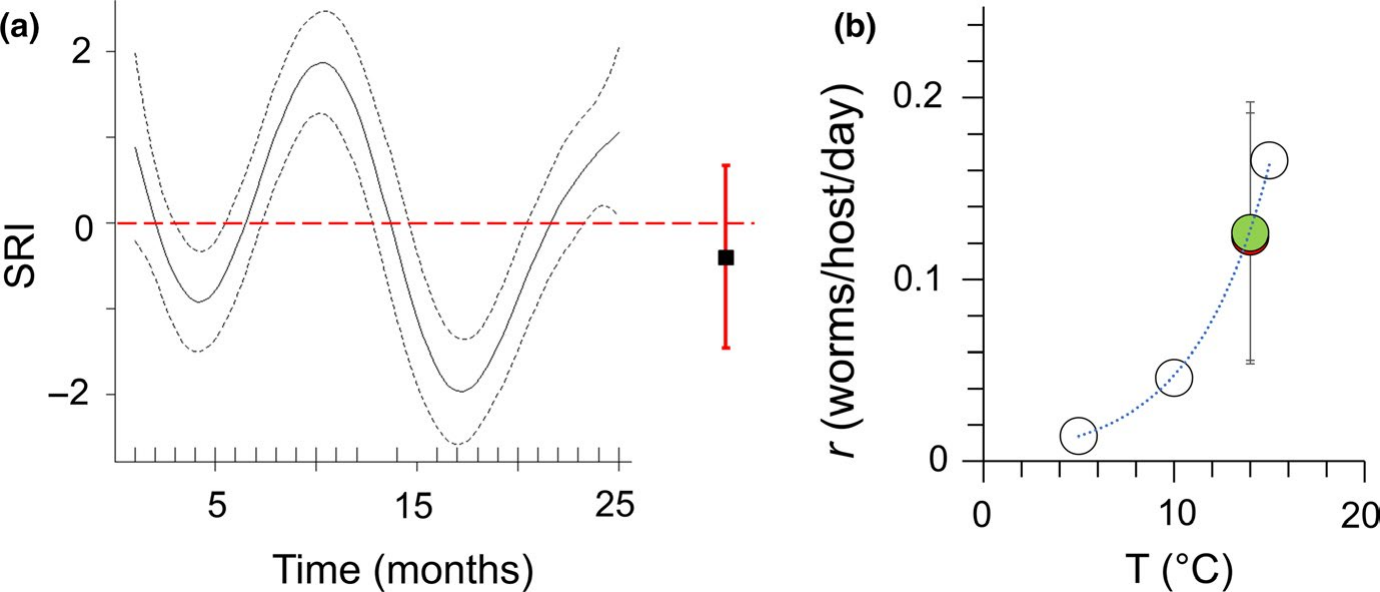
Power and effect size for the flow experiment, in the context of the known effects of other environmental variables. (a) Flow parameter estimate for immune‐associated gene expression (SRI) and 95% confidence interval (right) shown alongside smoothed SRI temporal (seasonal) variation (black line) in the field at RHD with 95% confidence interval (black dashed lines). The experiment and field SRI values are measured on the same scale, indicated for both by the y‐axis on the main plot. SRI (seasonal reporter index, SRI) is an additive index based on the expression of five separate genes (seasonal reporter genes, SR) known to reflect a consistent major seasonal oscillation in immune‐associated gene expression in wild stickleback (Brown et al., 2016; Stewart, Hablutzel, et al., 2018). (b) Least squares means for *Gyrodactylus gasterostei* intrinsic rate of increase (*r*) in the flow (red) and no‐flow (green) treatments of the challenge infection experiment (conducted at 14°C); bars indicating 95% confidence intervals. Open circles show *r* for *G. gasterostei* reported by Harris (1983) at different temperatures (*r* estimated by a regression method); a best fit exponential line is shown for reference

In the flume experiment, there was no overall effect of flow treatment on immune‐associated gene expression (PERMANOVA‐DM, *F*_1,50_ = 0.116, *p* = .967) or of infection stage (*F*_2,49_ = 1.682, *p* = .151) and no interaction between these (*F*_2,46_ = 0.581, *p* = .752). Nonetheless, a substantial association with sex was detectable (*F*_1,51_ = 8.930, *p* = .001, *R* ^2^ = 13.9%) and a modest association with condition (*F*_1,51_ = 4.509, *p* = .012, *R* ^2^ = 7.0%). Consistent with this, when analyzed individually, neither SRI nor any of the single gene expression variables were significantly associated with flow treatment, infection stage, or their interaction, but some associations were observed with length, condition, and especially sex (Table S1). To put possible effect size into perspective, the estimated flow parameter for SRI (negative for the zero‐flow treatment) is in the opposite direction to that required to drive the observed field circannual SRI fluctuation (where low flows coincide with high SRI). Furthermore, the upper 95% confidence limit for this parameter represents only ~ 18% of the smoothed temporal SRI range observed in the field (see also Figure 5). Thus, any undetected effect of countercurrent swimming on immune‐associated gene expression is likely to be at most small or negligible compared with other natural environmental variation.

For the *G. gasterostei* challenge infection, there was no significant effect of flow treatment on AUC^worm count^ (LM, *F*_1,32_ = 0.84, ns), peak intensity (LM, *F*_1,32_ = 1.29, ns) or *r* (LM, *F*_1,32_ = 0.002, ns; parameter = 0.002 ± 0.0486). To put possible effect size into perspective, the upper 95% confidence limit for the *r* flow parameter magnitude was equivalent to the rise in *r* due to a ~2.3°C rise in temperature from 14°C (the temperature of the current experiment), based on data reported by Harris (1983) (see also Figure 5). Thus, any undetected effect of countercurrent swimming is likely to be at most small or negligible compared to thermal effects.

## 4 DISCUSSION

Our study aimed to quantify the role of locomotor activity as a constraint on immune activity in wild fish. To achieve this, we matched long‐term records for water flow and immune gene expression in the wild with experimental estimates of the effect of sustained countercurrent swimming on immune gene expression and infection resistance in acclimated fish derived from a naturalistic outdoor habitat.

We initially observed that, in a natural lotic habitat across a 25‐month period, water flow was strongly correlated with temperature and stickleback immune‐associated gene expression (SRI). We have previously shown temperature to be a powerful causal driver of immune‐associated gene expression in sticklebacks (Stewart, Hablutzel, et al., 2018), and so we first asked if a direct effect of countercurrent swimming in response to flow could explain any variation over and above that explained by temperature variation. In this analysis, we assumed that any direct causal effect of flow on immune‐associated gene expression would be linear, that is, that increasing flow would result in proportional increases in countercurrent swimming, which in turn would drive a directional change in gene expression. In fact, we found no linear effect of flow on (thermally adjusted) immune‐associated gene expression (represented by SRI). Nonetheless, we recognized the possibility that a (real) flow effect might have been obscured in observational data (e.g., by confounding with temperature or an unmeasured environmental variable). We thus designed an experiment, using an open channel flume, to directly assess the effect of countercurrent swimming on immune‐associated gene expression.

In the flume experiment, acclimated wild fish were exposed to a water flow that stimulated rheotropism (countercurrent swimming). Sticklebacks are relatively weak, primarily labriform (Walker & Westneat, 2002), swimmers that readily seek refugia under conditions of high flow. Previous studies on sticklebacks from our study locality (RHD) suggest most individuals exhaust (cease to maintain station) within 8 hr at 20–30 cm/s (Whoriskey & Wootton, 1987). In our study, we empirically (following initial trials) set the current at 10 cm/s, allowing continuous swimming for an extended period (48 hr) which approached the limit at which some individuals would succumb to exhaustion. The experiment was thus intended to reflect relatively extreme conditions, maximizing the detectability of any locomotion effects. In the event, we found that there was no effect of sustained intense swimming on immune‐associated gene expression or infection resistance. Taken together with the lack of detectable linear flow effects in the field, we take this as strong evidence that locomotor activity negligibly constrains immune activity under natural conditions. It seems likely that in the wild, in practice, sticklebacks would exhaust (and likely die or be lost downstream), or seek flow refugia, before the effects of extreme physical activity on immunity were manifested.

Having ruled out an important direct effect of locomotory activity, it might further be considered whether flow itself has indirect effects in the wild. As we had not observed a (thermally adjusted) linear relationship between flow and immune‐associated gene expression (SRI) in wild fish, as would have been consistent with a direct flow effect via countercurrent swimming, we also addressed the possibility of a (thermally adjusted) nonlinear association. We found this nonlinear association was highly significant but only explained a modest additional increment of the variation seen in the wild fish (beyond that explained by temperature). The form of the association was such that higher levels of adaptive immunity occurred at low and high flows, adjusting for thermal effects.

One notional environmental driver of immunity that might follow a complex nonlinear trend with increasing flow would be water quality. In principle, for example, at low flows, chemicals and eutrophicating agents might inherently be concentrated by small water volume. Conversely, at high flows increased terrestrial run‐off or more powerful currents in river channels might mobilize certain substances more than normally. Other potential explanations could also be considered. Among these is an indirect influence of flow via effects on foraging, as we have recently shown strong diet effects on immunity (Friberg, Taylor, & Jackson, 2019). In this scenario, arthropod prey (which promote elevated adaptive immune activity) might be concentrated at low flows simply by the smaller volume of the river channel. In high flows such organisms, having some countercurrent locomotory capacity (Lancaster, 1999; Richardson, 1992; Sidler, Michalec, & Holzner, 2018), might accumulate in flow refugia (areas of slack water) alongside their predator. Another possibility is altered pathogen exposure according to flow regimen. For example, there might be increased transmission when fish are at high density due to low water volumes or when occupying confined refugia during high flows. These explanations (foraging or transmission effects) have the advantage of predicting gene expression in the observed direction (high adaptive gene expression, due to enhanced foraging for arthropods or increased pathogen transmission, at times of low and high flow).

An important caveat to the interpretation of the thermally adjusted statistical association between flow and immune gene expression is that the thermal adjustment does not exclude the possibility that some real (indirect) flow effects may be obscured by collinearity with temperature. If this were the case then flow may, overall, be a negative indirect driver of SRI (as the crude correlation is strongly negative), possibly with a substantial real effect size. In this eventuality, each of the feeding, pathogen exposure or hydrochemical explanations above might still be relevant—but with adverse hydrochemistry, pathogen pressure or poor feeding efficiency predominantly driving low adaptive immune activity at high flows. Although further testing of these hypotheses is beyond the scope of the present study, we nonetheless can infer that the effect size of any hydrochemical influence is unlikely to be large. Thus, water chemistry would be expected to vary seasonally and, if important, to drive different seasonal patterns in sites subject to very different hydrological conditions. In fact, we have recently reported (Stewart, Hablutzel, et al., 2018) similar seasonal patterns in SRI variation (tending to track temperature) in a lowland river (RHD), an upland lake, and even in mesocosms filled from mains water supply. Moreover, we did not detect any effect of experimental *G. gasterostei* infection on SRI (or individual gene expression variables) in the present study, suggesting “force of infection” in the wild is unlikely to drive seasonal SRI variation, although the effects of other common pathogens remain to be studied.

In summary, our key finding is that locomotory activity per se is unlikely to be an important constraint on immunocompetence in healthy sticklebacks under normal natural circumstances. The potential generality of this result is supported by the fact that sticklebacks are relatively poorly adapted to sustained rapid swimming and thus may be an especially sensitive system in which to detect locomotory effects on immunocompetence. In addition, we found that, despite the lack of a direct locomotory effect on immune expression, water flow was still statistically associated with the latter in wild sticklebacks; we conclude that this association must be driven by an unknown indirect mechanism.

## CONFLICT OF INTEREST

None declared.

## AUTHOR CONTRIBUTION

J.A.J. and J.C. applied for funding. N.M., J.C., and J.A.J. conceived the ideas and designed the methodologies. N.M. carried out the flume and infection experiments. R.S., P.I.H., and I.M.F. carried out molecular measurements. J.A.J. and N.M. analyzed the data. J.A.J., N.M., J.C., I.M.F., and P.I.H. contributed to writing the paper.

## Data Availability

The basic data from this study will be available in the European Nucleotide Archive (primary accession number PRJEB13319).
