# Supplementary material for: Not going with the flow: Locomotor activity does not constrain immunity in a wild fish
Source: Ecol Evol. 2019 Oct 2;9(21):12089–98. doi: 10.1002/ece3.5658 (PMC6854097; doi:10.1002/ece3.5658)
Supplement: Supplementary file 1 [file ECE3-9-12089-s001.pdf]

Figure S1

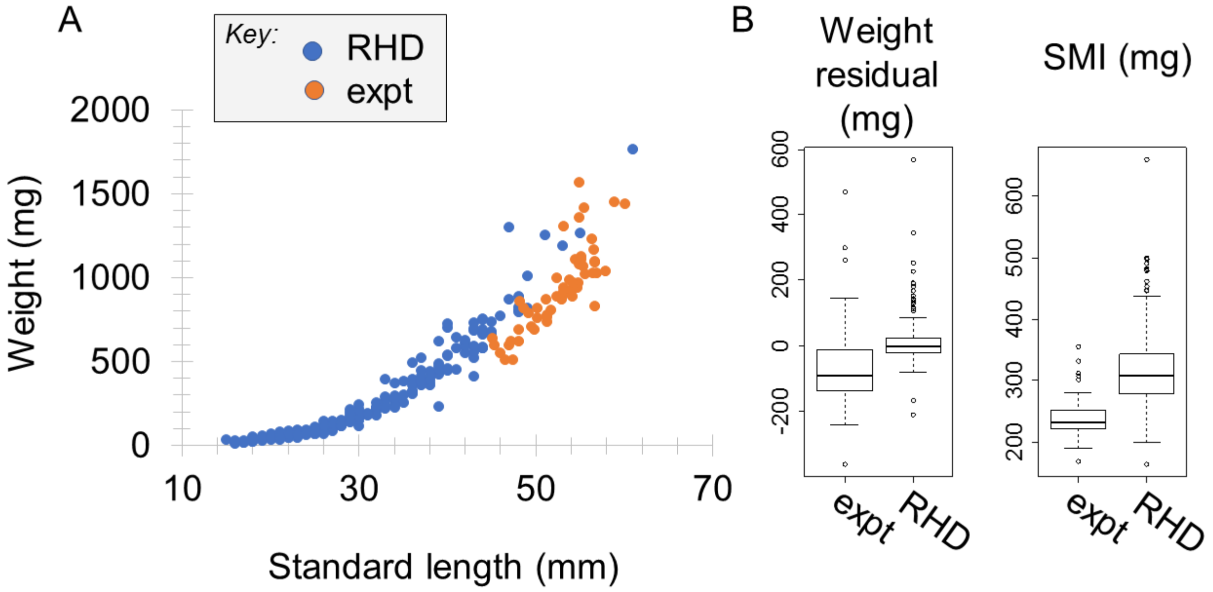

Acclimatized fish used in the flume experiment (expt) overlapped in size with and were, on average, in poorer condition than wild fish at RHD. **A**. Scatterplot of individual weight against standard length for wild fish at RHD (blue) and acclimatized fish from the flume experiment (orange). **B**. Box-and-whisker plots of the distribution of body condition measures in individual fish; showing median (middle line), second and third quartiles (box), range (whiskers), and outliers (points). Left-hand plot: weight residuals for individual fish, based on a quadratic regression of weight on standard length that included a term for sex and also interactions of sex with the linear and quadratic standard length terms (ANOVA, RHD 71.20 ± 14.11 mg, $P < 0.001$). Right-hand plot: scaled mass index, SMI (Peig & Green 2010) (ANOVA, RHD 76.24 ± 9.16 mg, $P < 0.001$).

Peig, J. & Green, A.J. (2010). The paradigm of body condition: a critical reappraisal of current methods based on mass and length. *Functional Ecology*, 24, 1323–32.
