# Supplementary material for: Not going with the flow: Locomotor activity does not constrain immunity in a wild fish
Source: Ecol Evol. 2019 Oct 2;9(21):12089–98. doi: 10.1002/ece3.5658 (PMC6854097; doi:10.1002/ece3.5658)
Supplement: Supplementary file 2 [file ECE3-9-12089-s002.pdf]

Table S1. Results (fixed effects) from linear mixed models (LMMs) analysing gene expression variation in the flume experiment. Each model initially included main effects for flow treatment, *Gyrodactylus* infection stage, sex, standard length, body condition and assay plate. Also included initially was an interaction term for flow treatment and *Gyrodactylus* infection stage (always non-significant and so removed) and random intercepts for flow trial (experiment batch) and RNA extraction batch. Unless non-significant (ns), results (parameter estimate ± standard error; *F* value and associated numerator and denominator degrees of freedom as subscripts; and *P* value) are presented for addition of the terms to a base model already containing terms for plate, flow trial and RNA extraction batch and all other significant fixed terms.

Gene expression variable	Flow	Infection stage	Sex	Length	Condition	plate
SRI	ns	ns	ns	ns	ns	ns
cd8a	ns	ns	ns	0.082 ± 0.035<br>$F_{1,53.4} = 5.52$<br>$P = 0.022$	ns	ns
foxp3b	ns	ns	male 1.121 ± 0.211<br>$F_{1,49.6} = 28.14$<br>$P = 2.62 \times 10^{-6}$	ns	ns	ns
ighm	ns	ns	male 0.782 ± 0.242<br>$F_{1,53.9} = 10.44$<br>$P = 2.1 \times 10^{-3}$	ns	0.003 ± 0.001<br>$F_{1,52.3} = 10.56$<br>$P = 2.0 \times 10^{-3}$	ns
orai1	ns	ns	male 0.782 ± 0.231<br>$F_{1,52.9} = 11.52$<br>$P = 1.3 \times 10^{-3}$	ns	0.003 ± 0.001<br>$F_{1,53.9} = 11.45$<br>$P = 1.3 \times 10^{-3}$	ns
tbk1	ns	ns	male 0.775 ± 0.247<br>$F_{1,46.5} = 9.81$<br>$P = 3.0 \times 10^{-3}$	ns	ns	ns
